# Targets and probes for non-invasive imaging of β-cells

**DOI:** 10.1007/s00259-016-3592-1

**Published:** 2016-12-26

**Authors:** Andreas Jodal, Roger Schibli, Martin Béhé

**Affiliations:** 1 Center for Radiopharmaceutical Sciences ETH-PSI-USZ, Paul Scherrer Institut, 5232 Villigen, Switzerland; 2 Department of Chemistry and Applied Biosciences, ETH Zurich, Zurich, Switzerland

**Keywords:** Imaging, β-cells, Pancreas, PET/SPECT, MRI, Optical imaging

## Abstract

β-cells, located in the islets of the pancreas, are responsible for production and secretion of insulin and play a crucial role in blood sugar regulation. Pathologic β-cells often cause serious medical conditions affecting blood glucose level, which severely impact life quality and are life-threatening if untreated. With 347 million patients, diabetes is one of the most prevalent diseases, and will continue to be one of the largest socioeconomic challenges in the future. The diagnosis still relies mainly on indirect methods like blood sugar measurements. A non-invasive diagnostic imaging modality would allow direct evaluation of β-cell mass and would be a huge step towards personalized medicine. Hyperinsulinism is another serious condition caused by β-cells that excessively secrete insulin, like for instance β-cell hyperplasia and insulinomas. Treatment options with drugs are normally not curative, whereas curative procedures usually consist of the resection of affected regions for which, however, an exact localization of the foci is necessary. In this review, we describe potential tracers under development for targeting β-cells with focus on radiotracers for PET and SPECT imaging, which allow the non-invasive visualization of β-cells. We discuss either the advantages or limitations for the various tracers and modalities. This article concludes with an outlook on future developments and discuss the potential of new imaging probes including dual probes that utilize functionalities for both a radioactive and optical moiety as well as for theranostic applications.

## Why is non-invasive β-cell imaging necessary?

The islets of Langerhans, dispersed all over the pancreas with the highest density in the tail, consist of different specialized cells: α-cells, responsible for the production of glucagon, δ-cells producing somatostatin, PP cells and ε-cells that secrete ghrelin. β-cells, making up to 65–80% of the cells in the islets, are involved in the control of blood glucose levels through insulin secretion [1–3]. Pathologic conditions disturb insulin secretion and lead to a blood glucose imbalance that strongly impairs quality of life and is potentially fatal if not treated. Hyperglycemia, which can result from insufficient insulin response, leads to long-term vascular damage and, in acute instances, can lead to life-threatening ketoacidosis [4]. Hypoglycemia, in contrast, can cause a critical lack of glucose in the brain, potentially resulting in cerebral damage or, in acute cases, neuroglycopenic symptoms and death [5]. The most prominent cause for hyperglycemia is diabetes, one of the most prevalent diseases of our time. It already affects more than 347 million people worldwide and the incidence continuously grows at an alarming rate [6]. Diabetes can be divided into two types. Type 1 diabetes is caused by an autoimmune reaction towards β-cells, resulting in a lack of insulin secretion, leading to hyperglycemia [7]. Type 2 diabetes, in contrast, is a chronic metabolic disease. In this case, the hyperglycemia is caused by the desensitization of the insulin receptors on different cell types, mainly in liver, muscle, and fatty tissue [8]. Initially, the β-cells can compensate for the lack of insulin response by an elevated insulin secretion level which, however, cannot be maintained in the long run and ultimately β-cells lose their function in response to factors like dedifferentiation or apoptosis [9, 10]. Since data about pathologic β-cells can only be obtained post mortem, a lot of the pathogenesis of diabetes is still under debate [1]. Currently, the standard diagnostic tests of both type 1 and 2 diabetes determine only indirectly if the patient is persistently hyperglycemic by analyzing either plasma glucose or glycated hemoglobin levels (HbA1c). However, these markers change only after more than 80% of the β-cells lost function. Thus, other alternatives are needed to monitor changes in β-cell function and mass [11].

Non-invasive β-cell imaging would allow following the pathogenesis of diabetes and might give more insight into the disease progression and β-cell mass as the disease develops. This could also eventually lead to a more efficient diagnostic method allowing physicians to tailor the treatment to the patient and to intervene before the loss of β-cell mass is irreversible. Additionally, β-cell imaging might allow in vivo tracking of transplanted islets, which in the future might be a prognostic tool to define treatment strategies for transplanted type 1 diabetes patients. Transplanted islets, focally engrafted into the brachioradialis muscle, were successfully labeled utilizing a radiolabeled GLP-1R agonist. Both the right transplantation and imaging protocols, however, have to be considered. A commonly used technique is the injection of the isolated islets into the portal vein, leading to the engraftment into the liver, as this results in a diffuse localization of the islets situated within an organ that non-specifically accumulates many tracers making a visualization very challenging [12–14].

Hypoglycemia, on the other hand, is a condition that can be caused by pathologic β-cells, which secrete an excess of insulin. The goal of the treatment of all kinds of hyperinsulinism is to reach normoglycemia. The standard treatment for insulinomas, the main reason for hyperinsulinism in adults, is resection. Treatment with drugs will be only performed if surgery is not an option for medical reasons. Non-invasive imaging of cancer cells, in contrast, allows the physician a precise assessment of the situation and specific excision of the affected tissue and therefore minimizes potential future complications.

The first-line approach for patients with congenital hyperinsulinism is medical therapy with diazoxide and later on with octreotide. Surgery is a second-line therapy if the medical application fails due to side effects or not responding, and if a focused lesion could be localized [15–17]. As both those foci as well as healthy β-cells are usually quite small, however, an imaging modality with both high sensitivity and resolution is required [18].

The majority of adult beta cell hyperplasia are non-focal hyperplastic manifestations and can be kept under control in most of the cases with medical treatment. Therefore, imaging may play a minor role in such patients.

**Fig. 1:**
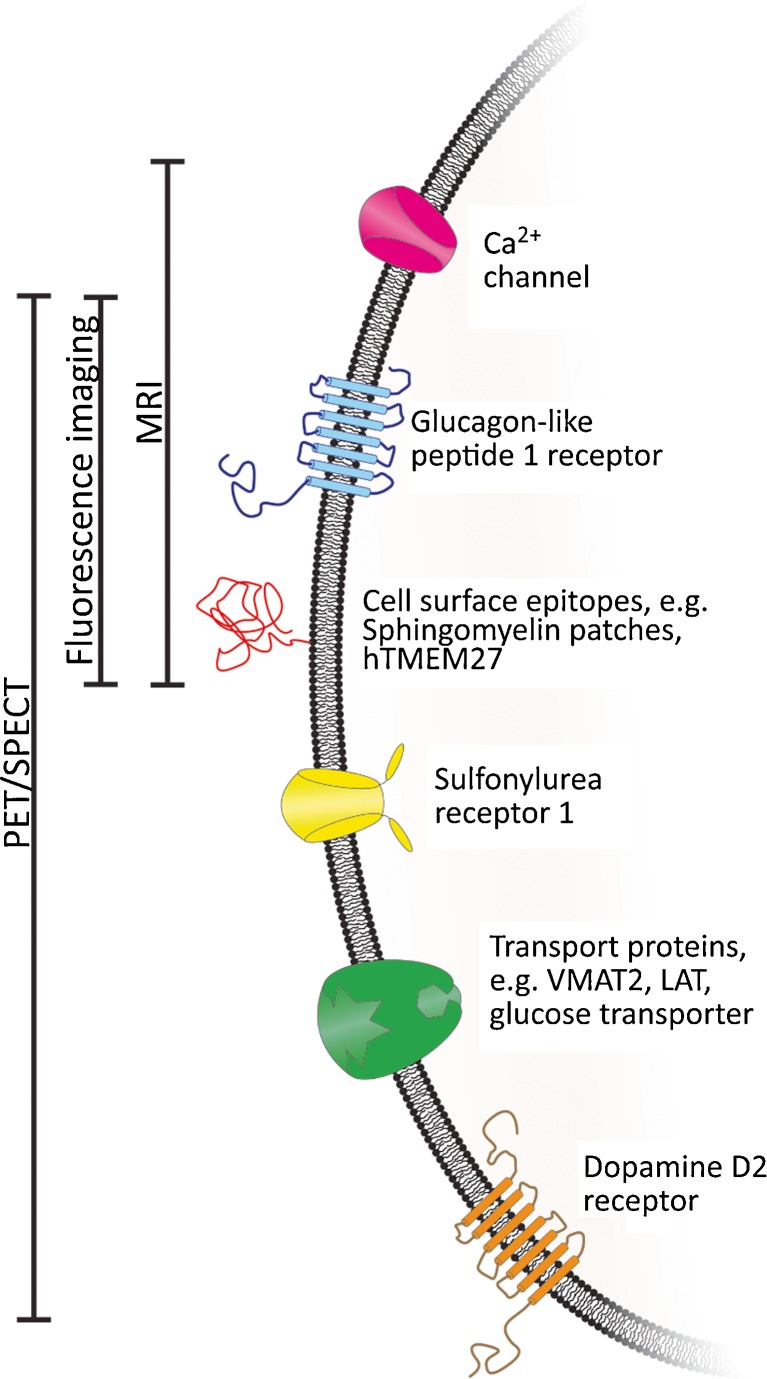
An overview of the most promising targets and imaging modalities reviewed in this article

This review gives a short overview of the various imaging modalities suitable for β-cell imaging. Techniques not offering a sufficient sensitivity or resolution to visualize pancreatic β-cells, such as computed tomography (CT) or diagnostic sonography, were omitted. Further, potential targets and probes that are currently being investigated as tracers for β-cell imaging are discussed with a special focus on radiotracers for positron emission tomography (PET) and single-photon emission computed tomography (SPECT). An overview of the possible targets is shown in Fig. 1 and Tables 1, 2.

**Table 1:** Advantages and disadvantages of the different imaging modalities covered in this review

	Sensitivity	Spatial resolution	Tissue penetration	Common tracer
PET	+	−	+	^11^C, ^18^F, ^68^Ga, ^64^Cu
SPECT	+	−	+	^99m^Tc, ^111^In, ^123^I, ^131^I
MRI	−	+	+	SPIO, Gd^3+^, Mn^2+^
Optical imaging	+	+	−	Luciferin, NIR fluorophores

**Table 2:** Summary of the tracer discussed in this review with the corresponding targets as well as their current status

Target	Imaging agent	Current status	References
VMAT2	[^11^C]DTBZ	Preclinical studies (in vitro and in vivo in rats)	[19]
	[^18^F]FE-DTBZ d4	Preclinical studies (in vitro and in vivo in pigs)	[20]
	[^18^F]-FP-(+)-DTBZ	Clinical studies	[21–23]
SUR1	^18^F and ^99m^Tc labeled glibenclamide derivatives	Clinical studies	[24]
	^99m^Tc-DTPA-glipizide	Preclinical studies (in vitro and in vivo in mice)	[25]
Sphingomyelin patches on the β-cell surface	^111^In-DTPA-IC2	Preclinical studies (in vitro and in vivo in mice)	[26, 27]
β-cell-surface epitopes	^125^I-labeled SCA B1, SCA B2, SCA B3, and SCA B4; SCA B2 functionalized carbon-coated cobalt NP	Preclinical studies (in vitro and in vivo in mice and rats)	[28, 29]
hTMEM27	AF 488 / [^89^Zr]-8/9-mAb	Preclinical studies (in vitro and in vivo in mice)	[30]
Glucose transporter	^18^F-FDG,	Clinical studies	[31, 32]
LAT	^18^F-DOPA, [^11^C]-5-HTP	Clinical studies	[33–37]
D2 receptor	[^18^F]-fallypride	Preclinical studies (in vitro and in vivo in rats)	[38–40]
Zn^2+^release	GdDOTA-diBPEN	Preclinical studies (in vitro and in vivo in mice)	[41–43]
Voltage-gated Ca^2+^ Ca^2+^ channels	Mn^2+^	Preclinical studies (in vitro and in vivo in mice), retrospective study in humans	[44–47]
Unclear	PiY	Preclinical studies (in vitro and ex vivo analysis of mouse organs)	[48]

## Non-invasive imaging modalities used for β-cell visualization

As β-cell islets are relatively small and occupy a minor portion of the pancreas, their imaging is challenging. Potential imaging modalities require certain properties, such as high sensitivity and spatial resolution, which limits the feasible choices. This chapter discusses the potential modalities and their properties and evaluates their advantages and shortcomings.

## Magnetic resonance imaging (MRI)

MRI uses strong oscillating magnetic fields to excite hydrogen atoms, mostly in tissues containing water molecules. Excited hydrogen atoms emit radio frequency signals that can be detected by a receiver and transformed into an image. As different tissues have a distinctive rate with which the excited hydrogen atoms return into the relaxed state, most anatomical structures can be clearly distinguished with a very high resolution. Sensitivity of MRI, however, is significantly lower compared to SPECT or PET, a concentration in the low micromolar range is necessary for detection [74–76]. Different contrast agents can be used to more clearly visualize specific tissues, the most common ones being gadolinium, iron oxide, and manganese [77].

### Single-photon emission computed tomography (SPECT)

The principle of SPECT is the administration of a γ-ray-emitting radiotracer. The signal is detected by rotating detectors registering the radioactive decay of the tracer in the subject’s body and can be reconstructed into a three-dimensional image. As the γ-emission is random and only the signal perpendicular to the detector is desired, a major fraction of the signal has to be filtered by collimators. Compared to most other imaging techniques, however, SPECT offers a high sensitivity in the picomolar range [75, 76, 78]. Commonly used radionuclides for SPECT are ^99m^Tc, ^111^In, ^123^I, and ^131^I [79]. One additional point that has to be taken into account is that only 1–2% of the pancreas are β-cells, meaning that the number of targets is limited. In consequence, the specific activity of the tracers targeting structures with limited quantities, like for instance β-cell receptors, is necessary to avoid saturation of the targets with non-active tracer, resulting in a strongly reduced signal [49]. Another minor challenge of SPECT imaging is the partial volume effect, which occurs if the signal originates from structures smaller than the resolution of the modality. This can lead to the underestimation of the signal and influences the quantification of β-cell mass (BCM). However, further analysis is required to determine to which extent the signal is affected.

### Positron emission tomography (PET)

PET, another nuclear imaging technique, detects the γ-rays resulting from the annihilation of a positron emitted from the radionuclide embedded in a tracer molecule. After collision with an electron, the resulting annihilation produces two photons that are emitted in a 180° angle and are detected by a detector ring. Events are only registered if they are a coincidence in an angle of 180°, eliminating the necessity for collimators and resulting in even higher sensitivity than SPECT in the femto- to picomolar range [76]. The time difference between the detection of the two photons allows an exact spatial localization of the disintegration permitting a three-dimensional reconstruction of the signal (time of flight; TOF) [80]. The most common radionuclide is ^18^F. Its physical and chemical features such as the low energy and high yield of the emitted β^+^ as well as the fact that it is a bioisostere of oxygen, allow easy replacement of hydroxyl groups. Therefore, it only slightly affects the properties of the molecule. Other frequently used PET nuclides are ^11^C, ^68^Ga, and ^64^Cu [81–83]. As for SPECT imaging, tracer aimed at β-cell-specific targets need to have a high specific activity. PET imaging also suffers from the partial volume effect.

### Optical imaging

Optical imaging is a growing field in preclinical and clinical applications. Different methods were developed including bioluminescence tomography (BLT), fluorescence molecular tomography (FMT), and optical projection tomography (OPT), which are described in detail in the review of Rzansky et al. [84]. Optical imaging in general describes the detection of light of a particular wavelength. Fluorescent proteins have been used for a long time to track cells and proteins in vitro. More recently, this technology has been applied for in vivo imaging in animals. The signal can be either generated by bioluminescence, where the optical moiety has to be activated in vivo by an enzyme, usually luciferase, which was transfected in the target tissue [85]. Fluorescent probes on the other hand contain an optical moiety that, after excitation at a specific wavelength, emits a signal of a different wavelength [86, 87]. In both cases, the optical signal with the defined wavelength can be detected with both a high spatial resolution and high sensitivity. Depending on the wavelength, the fluorescent signal is easily scattered. Thus, the imaging of organs is still challenging. A large variety of fluorophores are available, ranging from low wavelengths such as 510 nm (green fluorescence protein), which are not suitable for in vivo imaging, up to 900 nm (near-infrared flourophores), providing a better penetration for imaging of deeper tissues [86]. Such near-infrared (NIR) probes are successfully used for intraoperative detection of various lesions [88–90].

## Possible targets for radiotracers for β-cell imaging

### VMAT2

**Fig. 2:**
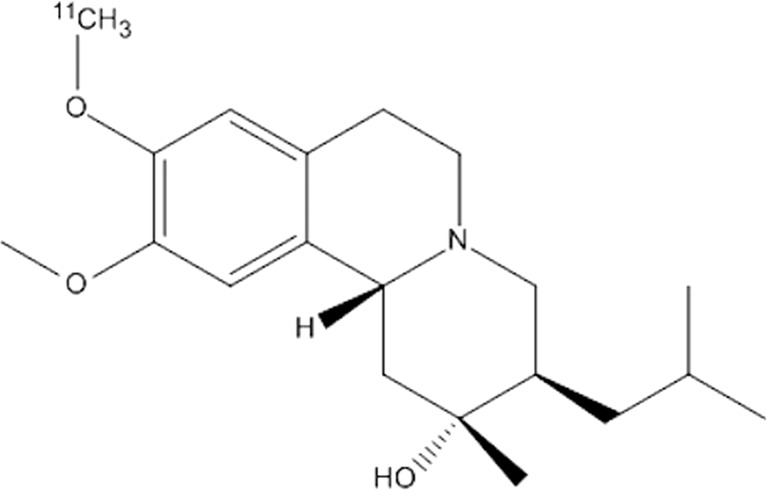
Structure of ^11^C-(+)-DTBZ as an example for VMAT2 tracer

The vesicular monoamine transporter 2 (VMAT2), an integral membrane protein responsible for the transport of neurotransmitters, is widely expressed in the central and peripheral nervous system but also in the hematopoietic and neuroendocrine systems. As an integral part of the neuroendocrine system, pancreatic β-cells express VMAT2, which is a promising target for PET/SPECT radiotracers [89, 91, 92]. So far, all studies have been performed with derivatives of dihydrotetrabenazine (DTBZ), the most prominent ones is [^11^C]DTBZ (Fig. 2) which was originally a tracer for diagnosis of patients suffering from Parkinson’s disease. [^18^F]fluoroethyl [FE]-DTBZ and the metabolically more stable deuterated analogue [^18^F]FE-DTBZ d4 are further promising analogues [19–21, 41–43, 93].

The applicability of these tracers, however, is under debate. According to Fagerholm et al., the highest activity after injection of [^11^C]DTBZ was found in the pancreas, yet the majority of the signal was not found in the islets but as non-specific binding to the exocrine pancreas as well as to PP cells in both human and rat samples. Similar findings were reported for [^18^F]FE-DTBZ in addition to a high metabolic defluorination, which led to a high ^18^F accumulation into the bones. The high uptake in PP cells and delta cells may be related to the expression of VMAT2 in these cells compared to β-cells [19, 21, 44–47]. Jahan et al. resolved the latter problem by the substitution of four ethyl hydrogens of [^18^F]FE-(+)-DTBZ to deuterium, resulting in the compound [^18^F]FE-(+)-DTBZ d4. However, the exocrine-to-islet ratio remained the same, leading to the conclusion that BCM determination might not be feasible, but the tracer might be used for studying focal clusters of β-cells, as in the case of intramuscular transplantation [20, 48]. In addition, Normandin et al. reported quantifiable changes in BCM of type 1 diabetic patients, which is not observed in healthy humans after the administration of ^18^F-FP-(+)-DTBZ. Furthermore, the non-specific binding was not determined, and it is not clear if BCM might have been overestimated [22, 49–51]. A study in baboons, performed by Harris et al., tried to quantify the non-specific binding in the pancreas using ^18^F-FP-(−)-DTBZ, the negative enantiomer that does not bind to VMAT2. Subtracted from the binding of ^18^F-FP-(+)-DTBZ, it should give the specific β-cell related signal. Even though the results are promising, some critical factors remain. First, the non-specific binding in diabetic patients was higher due to persistent inflammatory changes. Secondly, δ and PP cells can also express VMAT2 adding to β-cell signals. Thus, these two factors contribute to the overestimation of BCM in patients with type 1 diabetes [23, 52, 89].

An additional important point regarding targeting VMAT2 is the appropriate animal model. Many in vivo studies were performed in rodent models [19, 43, 53, 93, 94]. However, Schäfer et al. showed that rodent species do not express VMAT2 in β-cells. The observed uptake in the pancreas may be caused by binding to VMAT2 expressed in sympathetic nerve terminals that innervate the islets and mast cells. Models suitable for β-cell-specific VMAT2 imaging would be non-human primates and pigs, as they show similarly high VMAT2 expression as humans [43, 95].

In conclusion, VMAT2 might be a useful target for β-cell imaging. Current tracers still display a high unspecific uptake, mainly in the exocrine pancreas, which has to be further optimized. In addition, it is important to apply an appropriate in vivo model as rodents do not express VMAT2 in the islets.

### SUR1

**Fig. 3:**
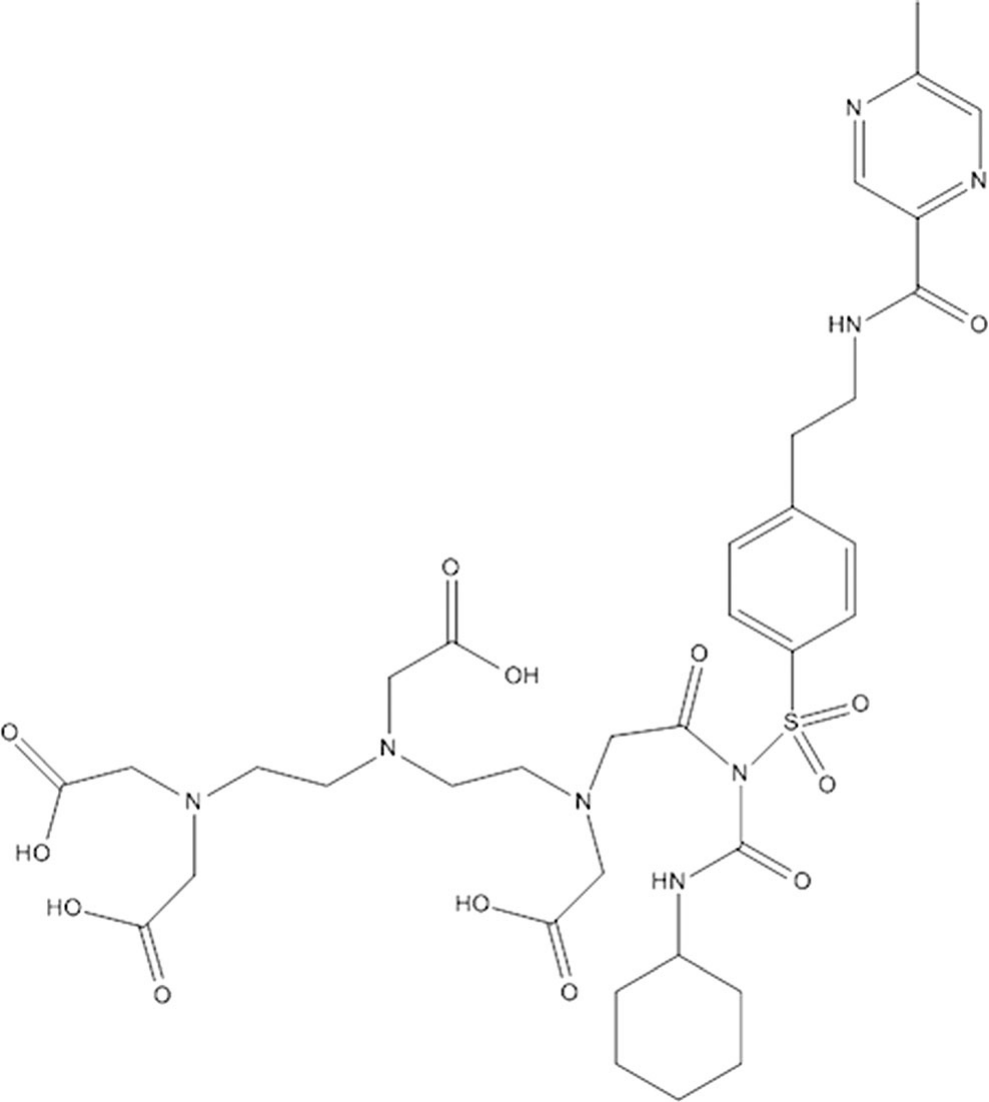
Structure of DTPA-glipizide, a tracer that can be used for the visualization of SUR1

The sulfonylurea receptor 1 (SUR1) is a subunit of ATP-dependent potassium channels (K_ATP_), which are involved in the insulin secretion from β-cells. While different isoforms of SUR exist, SUR1 is specifically found on β-cells and in the brain. Sulfonylureas like tolbutamide, glibenclamide, and glipizide, are widely used drugs for the management of type 2 diabetes [33, 49–51, 54, 96]. So far, a few radiolabeled SUR1 derivatives have been tested as potential candidates for β-cell imaging. Schneider et al. investigated various ^18^F and ^99m^Tc labeled glibenclamide derivatives. Although good binding affinity to the receptor as well as pharmacological effects were observed, an imaging study in humans was not successful. A stable radioactive signal was detected in the pancreas, however, a high background hindered reliable quantification [24, 55]. ^99m^Tc-DTPA-glipizide (Fig. 3), another sulfonylurea derivative assessed by Oh et al., was tested in mice. Due to its higher hydrophilicity, the main accumulation of the tracer changed from liver and intestine to the kidneys. Even though a persistent accumulation in the pancreas was detectable, the specific signal was still very low in comparison to the background [25, 56].

Currently, none of the available probes targeting SUR1 are feasible for β-cell imaging. Thus, further studies are necessary to improve SUR1-targeting traces.

### Other β-cell-surface epitopes

Various antibodies aimed at β-cell-specific epitopes have recently been tested. IC2, an antibody directed against sphingomyelin patches present on the β-cell surface, is one potential tracer that could be utilized for imaging β-cells. So far, it was shown that the antibody specifically binds to both human and murine β-cells and that the uptake of ^111^In-DTPA-IC2 correlates with β-cell mass in healthy and diabetic mice [26, 57, 97]. In another study, Ueberberg et al. used a phage library to generate highly specific single-chain antibodies against human β-cells. After administration of ^125^I-labeled antibody, the pancreatic uptake of the tracer with BCM correlates, but more extensive in vitro and in vivo studies are needed to fully assess the promising potential of those antibodies [28, 58].

The transmembrane protein 27 (TMEM27), another potential antigen, is specifically expressed on the β-cell surface and on kidney collecting ducts. Vats et al. characterized 8/9 mAb, an antibody against the human variant of TMEM 27 (hTMEM27). They used both a fluorescent labeled and radiolabeled construct to determine the uptake in tumor xenografts and islets of transgene mice. The tracers showed a high and specific uptake into hTMEM27-positive tumors after 1 day. Uptake in pancreatic sections co-localized with insulin staining, confirming the specificity for β-cells. Nevertheless, the sensitivity of the antibody has to be improved as the expression of hTMEM27 was ten times lower in humans as compared to the transgenic mice used in this study [30, 59].

These encouraging results for β-cell imaging with antibodies requires further validation studies such as biodistribution analysis and PET or SPECT scans in relevant animal models. Furthermore, a potential disadvantage of using antibodies as radiotracer includes long blood circulation time leading to a longer exposure and potentially higher radiation burden. Additionally, as they are highly lipophilic, most antibodies accumulate in the liver, potentially adding to the background.

### Radiolabeled saccharides and neurotransmitters

Tracers that are not specific for β-cells have also been used to image various β-cell-related conditions. ^18^F-FDG, the most prominent PET tracer, was successfully used to visualize transplanted islets. A small fraction of the islets were labeled ex vivo with ^18^F-FDG prior to intraportal transplantation and mixed with the rest of the islets. Eriksson et al. were able to assess the distribution of the transplanted islets in five patients for an hour. The short half-life (110 min) of ^18^F, however, limits its use for long-term studies [31, 49–51, 60].

A study performed by Malaisse et al. evaluated the uptake of ^18^F-FDG in healthy and streptozotocin-induced diabetic rats. In this study, they failed to see a lower accumulation in the diabetic pancreas compared to the control, leading to the conclusion that ^18^F-FDG is not suitable for the detection of pancreatic β-cells [32, 61].

**Fig. 4:**
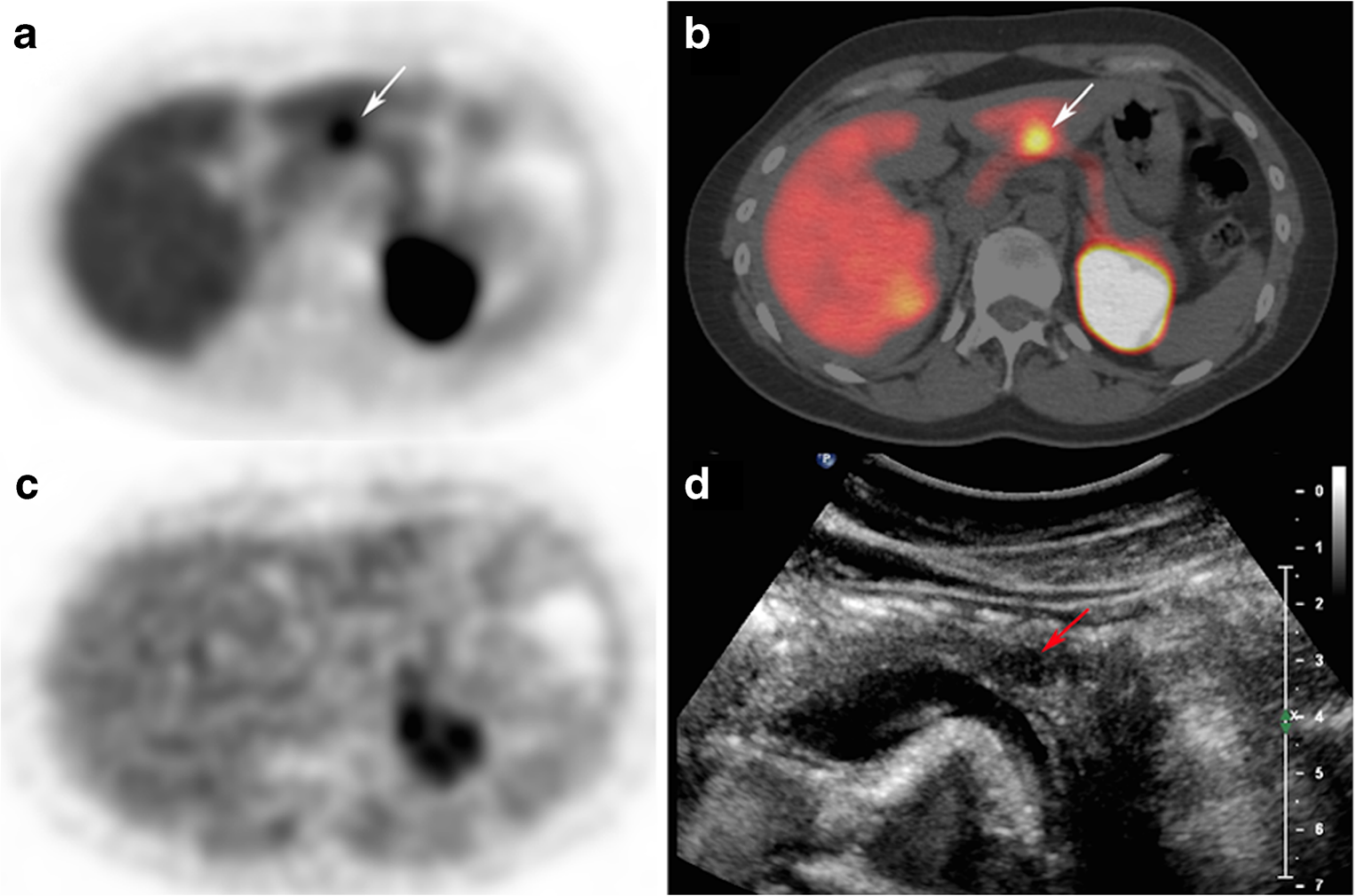
Carbidopa-assisted ^18^F-DOPA PET of a patient suffering from insulinoma. The* arrow* clearly indicates the lesion. **a** Early PET acquisition. **b** Axial PET/CT fusion. **c** Delayed PET acquisition. **d** Contrast-enhanced ultrasonography [33]

^18^F-l-dihydroxyphenylalanine (^18^F-DOPA), a metabolic tracer like FDG, is taken up by the large amino acid transporter (LAT) and metabolized to ^18^F-dopamine by the DOPA decarboxylase trapping it in the cells. Since it taken up in both exocrine and endocrine pancreatic cells, the potential use is limited. Still, patients suffering from hyperinsulinism, either caused by insulinomas or β-cell hyperplasia, can benefit from imaging with ^18^F-DOPA. Several groups have reported successful imaging of those foci [34, 35, 62, 63]. A report from Tessonier et al. in contrast reports an underestimation of the extent the disease after ^18^F-DOPA imaging. Nevertheless, it seems that the pancreas has high decarboxylase activity, leading to a high background [64–67, 98]. Imperiale et al. suggest pre-administration of carbidopa, which lowers the pancreatic ^18^F-DOPA activity while preserving the signal in the foci (Fig. 4) [19, 33, 68–70]. [^18^F]-fallypride, a dopamine D2-receptor antagonist, first synthesized by Mukherjee et al., is commonly used as a PET brain tracer [41, 99]. With the discovery that the D2-receptor is also expressed in β-cell islets where it co-localizes with insulin granules, however, it was suggested to use the receptor as a target for β-cell imaging [38, 60]. Garcia et al. confirmed the uptake in vivo. Imaging of the pancreas, though, proved to be difficult. On the one hand, the uptake in the islets was significantly lower than in the brain; on the other hand, the unspecific binding in the islets was approximately 50%. Nonetheless, a significant reduction of the pancreatic uptake was observed in streptozotocin-induced diabetic rats, which correlated with the loss of the β-cells. Furthermore, Garcia et al. were able to localize human islets pre-labeled with [^18^F]-fallypride, 1.5 h after transplantation using PET [39, 40, 71].

[^11^C]-5-Hydroxytryptophane ([^11^C]-5-HTP), originally a tracer to determine serotonin biosynthesis in various tissues is nowadays clinically used to diagnose neuroendocrine tumors like insulinoma. Similarly to previous tracers, [^11^C]-5-HTP is taken up by the large amino acid transporter (LAT) and specifically metabolized by the DOPA decarboxylase to [^11^C]-serotonin, which is trapped intracellularly. Various studies have shown that the islets of Langerhans, particularly the granules of the β-cells, specifically accumulate serotonin, making [^11^C]-5-HTP a potential tracer to image β-cells [42, 100, 101]. Even though [^11^C]-5-HTP also accumulates in other endocrine islet cells, Ericsson et al. were able to distinguish between healthy and type 1 diabetic patients as the tracer accumulation was strongly reduced [36]. Di Gialleonardo et al. in contrast were not able to differentiate between endocrine and exocrine pancreas, however, as they only performed in vitro assays on cell lines, additional experiments would be necessary to test this hypothesis in vivo [28, 29, 37, 68, 70]. Overall, [^11^C]-5-HTP seems to be a promising tracer, especially for imaging insulinomas and transplanted islets. However, it will not be able to distinguish between healthy subjects and subjects with diabetes due to a too large overlap between the groups.

### GLP-1 receptor

**Fig. 5:**
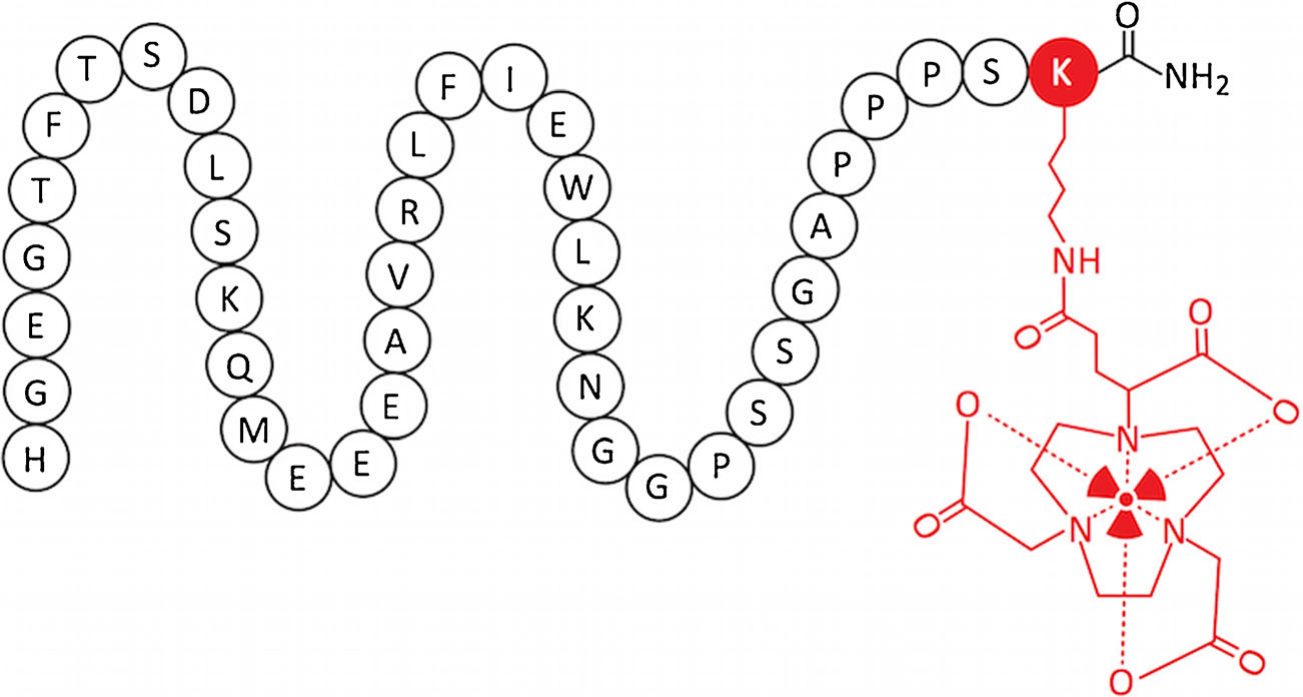
Structure of Ex4NOD40 as an example of radiolabeled exendin-4 derivatives. The moieties in* red* have been attached to the C-terminal end of the peptide

**Fig. 6:**
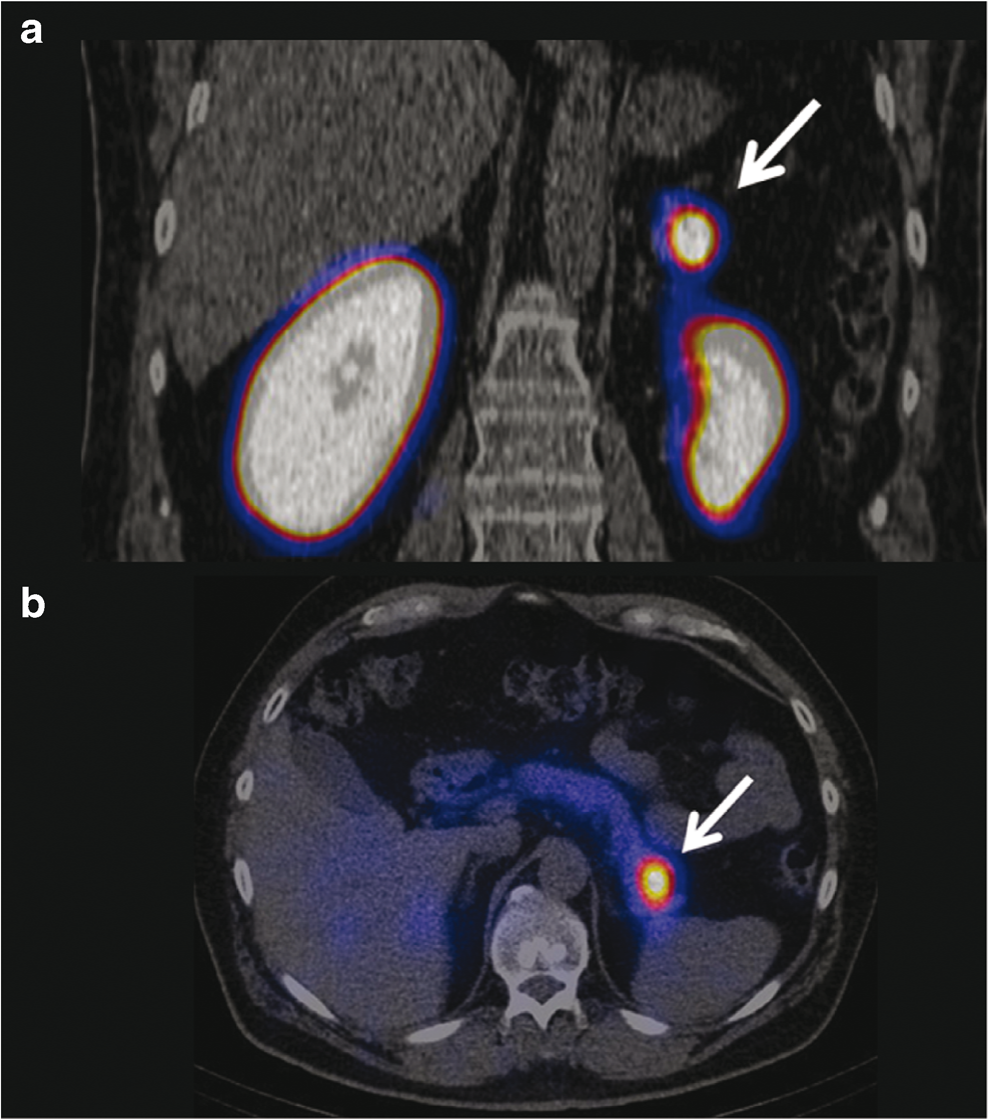
Coronal (**a**) and transaxial (**b**) SPECT/CT images from a patient with biochemically proven hyperinsulinemic hypoglycemia 72 h after the injection of 85 MBq ^111^In-DOTA-exendin-4. There is focal uptake of ^111^In-DOTA-exendin-4 in the pancreatic tail (*arrow*) consistent with the surgically removed and histological proven insulinoma

The glucagon-like peptide 1 receptor (GLP-1R), expressed in β-cells, stimulates insulin synthesis and secretion as well as promotes β-cell proliferation. As it is specifically expressed on β-cells, it is a viable target for imaging [73, 102–107]. The endogenous peptide GLP-1, however, has a very short plasma half-life, as it is rapidly metabolized by dipeptidyl peptidase-4 (DPP4), making it unsuitable as a tracer [33, 108]. Exendin-4, in contrast, a peptide first isolated from the saliva of *Heloderma suspectum*, is highly stable in vivo while having the same high affinity to the receptor as GLP-1 and induces the insulin secretion as well [47, 103, 107]. Due to the high affinity and efficacy synthetic exendin-4, known as exenatide, is successfully used for the treatment of type 2 diabetes. These pharmacological properties qualify exendin-4 as a lead compound for the tracer development for β-cell imaging, such as the C-terminally modified peptide (Fig. 5). Gotthardt et al. showed that ^111^In-DTPA-Lys^40^-exendin-4, a radiolabeled derivative of exendin-4, specifically binds to GLP-1R-positive tissues in rats. In addition, they were able to successfully image those structures with SPECT, concluding that this imaging technique might be used to examine both healthy GLP-1R-expressing tissue as well as tumors [64, 70]. Wild et al. confirmed this hypothesis using the same exendin-4 derivative in Rip1Tag2 transgenic mice with spontaneous insulinoma showing exceptionally high uptake in the tumors [50]. Recent studies in human patients confirmed the potential for the pre- and intraoperative detection of insulinomas (Fig. 6). The results indicate an improvement compared to currently used diagnostic methods for non-malignant insulinomas, as it is more sensitive than CT/MRI. The authors examined three patients with β-cell hyperplasia during this study and concluded that the detection of β-cell hyperplasia with radiolabeled exendin may be insufficient because the large interindividual variation of β-cells [51, 52]. Further studies to clarify the value of GLP-1 receptor imaging in patients with β-cell hyperplasia are necessary.

**Table 3:** Summary of the GLP-1R-specific tracer reviewed in this article and their current status

Tracer	Current status	References
[Lys^40^(Ahx-DTPA^111^In)NH_2_]-exendin-4	Clinical studies	[49–51]
Lys^40^(Ahx-^111^In-DOTA)NH_2_ exendin-4	Clinical studies	[52]
[Lys^40^ (Ahx-HYNIC-^99m^Tc/EDDA)NH_2_]-exendin-4	Clinical studies	[43, 53]
[Lys^40^(Ahx-DOTA-^68^Ga)NH_2_]-exendin-4	Preclinical studies (in vitro and in vivo in mice)	[43]
^68^Ga-DO3A-Exendin-4	Preclinical studies (in vivo in mice and non-human primates)	[33, 49–51, 54]
^64^Cu-DO3A-VS-Cys40-exendin-4	Preclinical studies (in vitro and in vivo in mice)	[55]
^64^Cu/ ^68^Ga-[Nle^14^,Lys^40^(Ahx-NODAGA)NH_2_]-exendin-4	Preclinical studies (in vitro and in vivo in rats)	[56]
[^18^F]FBEM-[Cys^x^]-exendin-4	Preclinical studies (in vitro and in vivo in mice)	[57]
^18^F-TTCO-Cys^40^-exendin-4	Preclinical studies (in vitro and in vivo in mice)	[58]
^18^F-E4_Tz12_	Preclinical studies (in vitro and in vivo in mice)	[59]
[^18^F]Ex(9–39)	Preclinical studies (in vitro and in vivo in rats)	[49–51, 60]
[^18^F]AlF-NOTA-MAL-cys^40^-exendin-4	Preclinical studies (in vitro and in vivo in mice)	[61].
^125^I-GLP-1(7–36), ^125^I-BH-exendin(9–39)	Preclinical studies (in vitro and ex vivo in autoradiography in mouse and human tissue)	[62, 63]
[Lys^40^(^111^In-DTPA)]-exendin-3	Preclinical studies (in vitro and in vivo in mice and rats)	[64–67]
[Lys^40^(^111^In-DTPA)]-exendin-4		
[Lys^40^ (^111^In-DTPA)]-exendin-4 (9–39)		
Lys^40^(^68^Ga-DOTA)]-exendin-3		
Lys^40^(^111^In-DOTA)]-exendin-3		
^68^Ga-Ex4NOD12 ^68^Ga-Ex4NOD27 ^68^Ga-Ex4NOD40	Preclinical studies (in vitro and in vivo in mice)	[68–70]
^125^I -Liraglutide	Preclinical studies (in vitro and in vivo in mice)	[41]
[^64^Cu]NODAGA-MAL-exendin-4	Preclinical studies (in vitro and in vivo in rats)	[60]
^111^In-PSI-CLNOD1	Preclinical studies (in vitro and in vivo in mice)	[71]
^111^In-PSI-CLNOD2		
^111^In-PSI-CLNOD3		
MN-Ex10-Cy5.5	Preclinical studies (in vitro and in vivo in mice)	[42]
Np647–ExCys1	Preclinical studies (in vitro and in vivo in mice)	[72]
E4_K12_-Fl E4_X12_-VT750	Preclinical studies (in vitro and in vivo in mice)	[68, 70]
^64^Cu-E4-Fl	Preclinical studies (in vitro and in vivo in mice)	[73]

To date, various exendin derivatives have been tested with the goal of either utilizing different nuclides or changing the pharmacological properties. [Lys^40^ (Ahx-HYNIC-^99m^Tc/EDDA)NH_2_]-exendin-4, another SPECT tracer utilizing the widely available ^99m^Tc, also successfully visualized insulinomas in vivo with the added benefit regarding the estimated effective dose, which was 40 times lower as compared to ^111^In labeled lead compound. A first in man study confirmed the usefulness of this tracer in imaging benign insulinoma foci in patients [43, 53]. [Lys^40^(Ahx-DOTA-^68^Ga)NH_2_]-exendin-4 on the other hand proved to be a potential alternative to [Lys^40^(Ahx-DTPA-^111^In)NH_2_]-exendin-4 for PET imaging, potentially allowing the localization of smaller insulinomas due to the superior resolution of PET while retaining the pharmacokinetics. In addition, the radiation burden of ^68^Ga is also lower than for the ^111^In, minimizing the dose for the patients [43]. Selvaraju et al. performed a study with a similar peptide, ^68^Ga-DO3A-exendin-4 in rats as well as in non-human primates. The difference of tracer uptake in the pancreas after streptozotocin-induced destruction of β-cells was observed, suggesting that a non-invasive quantification of GLP-1R is feasible [54]. A comparison between ^68^Ga and ^64^Cu revealed that [^64^Cu]NODAGA-exendin-4 shows a higher specific uptake in GLP-1R-expressing tissue than the ^68^Ga-labeled peptide in rats. PET imaging, however, failed to visualize the pancreas. Kirsi et al. concluded that the high radiation burden due to the high kidney uptake limits the feasibility of the ^64^Cu-labeled peptide as a clinical tracer [56]. Another ^64^Cu-labeled peptide, DO3A-VS-Cys40-exendin-4 tested by Wu et al. strongly accumulated in INS-1-grafted tumors in NOD/SCID mice and successfully visualized transplanted islets [55].

Two ^18^F exendin-4-based tracer [^18^F]FBEM-[Cys^x^]-exendin-4, derivatized either at the C-or N-terminal end of the peptide, tested by Kieswetter et al., showed high tumor uptake where the C-terminally modified derivative seemed to have a superior tumor uptake and tumor-to-background ratio. ^18^F-TTCO-Cys^40^-exendin-4 and ^18^F-FBEM–Cys^39^-exendin-4, two similar derivatives tested by Wu et al. and Xu et al., respectively, showed comparable results [57, 58]. Other ^18^F-labeled tracers were either modified on the lysine in position 12 (^18^F-E4_Tz12_) or position 27 ([^18^F]Ex(9–39)) and modified ^18^F-E4_Tz12_ shows similar distribution as original peptides,. A comparison of PET images between diabetic and non-diabetic rats did not yield any difference [59, 109]. Nevertheless, the question remains if this method is sensitive enough.

A disadvantage of covalently bound ^18^F tracers is the complicated and time-consuming labeling process of those peptides. In contrary [^18^F]AlF-NOTA-MAL-cys^40^-exendin-4 can be easily synthesized and retains the high uptake in GLP-1R expressing tissue. Unfortunately, the kidney uptake of [^18^F]AlF-NOTA-MAL-cys^40^-exendin-4 is much higher than the peptides with covalently bound ^18^F [61].

Studies with exendin-4(9–39) derivatives, which are GLP-1R antagonists can also potentially be used to image β-cells. Brom et al., however, conclude that agonists are more favorable, as they accumulate stronger in the tumor, most probably because of the higher internalization of the agonist. Waser et al., in contrast, suggest that for insulinoma, antagonists might be favorable, as they do not induce a transient hypoglycemic effect as opposed to GLP-1R agonists [63, 66, 109].

A study by Jodal et al. investigated the influence on different potential conjugation sites of exendin-4 labeled with ^67/68^Ga, concluding that the lysines in position 12 and 40 have the least influence on the biological properties of the peptides. The lysine in position 27, though, is also potentially modifiable without losing too much affinity, which is in contrast to findings of Wang et al. who did not see any remaining specific binding of ^18^F-labeled exendin-4(9–39) conjugated to Lys 27, leading to the conclusion that depending on the modification, the binding to the receptor might be impaired.

Another peptide tested by Brom et al. is exendin-3, which differs in only two amino acids from exendin-4. Lys40(^68^Ga-DOTA)] exendin-3 successfully visualized INS-1 tumor xenografts in BALB/c nude mice. ^111^In labeled Lys^40^(DTPA)]-exendin-3 showed comparable biodistribution to the corresponding exendin-4 derivative. This peptide was successfully used to quantify BCM in rats. However, studies in humans confirmed high interindividual differences in BCM, which is in line with previous reports. Nevertheless, pancreatic uptake of the tracer in diabetic patients was reduced as compared to healthy subjects, indicating that this might be a feasible technique for BCM quantification in humans [66, 67].

Recently, ^125^I-labeled liraglutide, a stabilized derivative of the natural ligand GLP-1, was assessed as a probe. The binding affinity of the peptide was lower than for previously tested exendin derivatives and even though INS-1 tumors were visualized in a xenograft mouse model, background activity was very high with much of the activity accumulating in non-GLP-1-positive organs like liver, thyroids, and salivary glands [41].

Therapeutic studies revealed that application of up to 28 MBq [Lys^40^(Ahx-DTPA^111^In)NH_2_]-exendin-4 leads to a significant reduction of the tumor size of up to 94%, while healthy β-cells were not affected. One issue, however, is a high, non-GLP-1R-mediated uptake in the kidneys, resulting in morphological changes after the administration of 28 MBq tracer. This limits the applicability of Lys^40^(Ahx-DTPA^111^In)NH_2_]-exendin-4 as a therapeutic agent [49]. The same observation was made by Velikyan et al. for the therapy with ^177^Lu-labeled DO3A-VS-Cys(40)-exendin-4 [110]. A high kidney uptake is a common issue with exendin derivatives, not just because of the radiation burden but also the high background, which might hamper the detection of a specific signal in close proximity to the kidneys. The latter is especially an issue for the imaging of non-hyperplastic tissues, as the uptake is less concentrated, leading to a lower signal intensity [53, 56, 69]. Various attempts have been made to reduce the kidney accumulation. One clinically applied method is the pre-administration of amino acids. Gotthardt et al. applied this method for exendin and tested the kidney uptake after the administration of lysine, gelofusine, polyglutamic acid, or the combination of gelofusine and polyglutamic acid. While the administration of lysine did not have any affect, both gelofusine and polyglutamic acid as well as the combination of both had an significant impact on the uptake of exendin in the kidneys [111]. A different approach to reduce kidney uptake is the introduction of a cleavable linker before the radiolabeled moiety of the peptide. This linker should be specifically cleaved at the kidney brush-border membrane, liberating the radioactive moiety as a small fragment that can be easily excreted in the urine. An *N* ^ε^-maleoyl-l-lysine linker, cleaved by various peptidases on the brush-border membrane of kidney proximal tubule cells has been successfully used to reduce kidney uptake of fab fragments [112]. Yim et al. adapted this linker for exendin-4, however, the kidney uptake of [^64^Cu]NODAGA-MAL-exendin-4 was not improved compared to the reference substance [60]. Another peptidase that could be potentially used to specifically cleave linker sequences is meprin β. Jodal et al. designed three exendin-4 derivatives containing liner sequences specific for meprin β. While those linkers were cleaved in vitro, they did not manage to reduce the kidney uptake in vivo [71].

These results show that the GLP-1R is a very promising target for the assessment of β-cells and can be utilized for different purposes. Many different exendin derivatives have been tested so far, proving the versatility of this peptide and making it the most viable option for GLP-1R targeting up to now. One remaining challenge, however, is the high kidney accumulation, which potentially can lead to a high radiation burden for the patient.

## β-cell visualization with MRI and optical imaging

### Magnetic resonance imaging (MRI)

As β-cells and insulinoma are small structures, a modality with high spatial resolution would be ideal to visualize individual islets or clearly defined foci. Utilizing the superior spatial resolution of MRI, Balla et al. successfully managed to image single islets, both ex vivo and in vivo using carbon-coated ferromagnetic cobalt nanoparticles functionalized with a β-cell-specific single-chain antibody as a contrast agent. Nevertheless, some issues remain unsolved. On the one hand, it unspecifically accumulated in liver and spleen, which is a common problem for most nanoparticles. On the other hand, potential cytotoxic effects of the nanoparticles have to be investigated before studies in humans can be conducted [29]. Various functionalized and unfunctionalized nanoparticles have been tested so far (Table 3), most of them, however, only to label transplanted islets [113–116]. Wang et al. and Vinet et al. both assessed nanoparticles that have been functionalized with exendin-4 targeting GLP-1R in mice. Both report a significant reduction of the contrast agent in diabetic mice compared to uptake in healthy controls, concluding that they might be of use in monitoring the development of diabetes [72, 117]. Long-term toxicity studies of nanoparticles, however, are still missing, and have to be performed before a clinical application.

A different approach utilizes the fact that insulin is stored in combination with Zn^2+^ in vivo, meaning that the extracellular concentration of Zn^2+^increases after the glucose-dependant release of insulin. After administration of the Zn^2+^-responsive contrast agent GdDOTA-diBPEN, Lubag et al. were able to detect a pancreatic signal specifically in healthy mice after administering glucose. In both streptozotocin-induced diabetic mice and healthy controls that did not receive glucose, no pancreas signal was detected. An increased uptake was found in mice that were on a high-fat diet for an extended period. This is consistent with previous reports on increased insulin secretion in obese, insulin-resistant patients [42]. Nevertheless, more studies are necessary to see if it is possible to differentiate between the change in BCM and β-cell function.

**Fig. 7:**
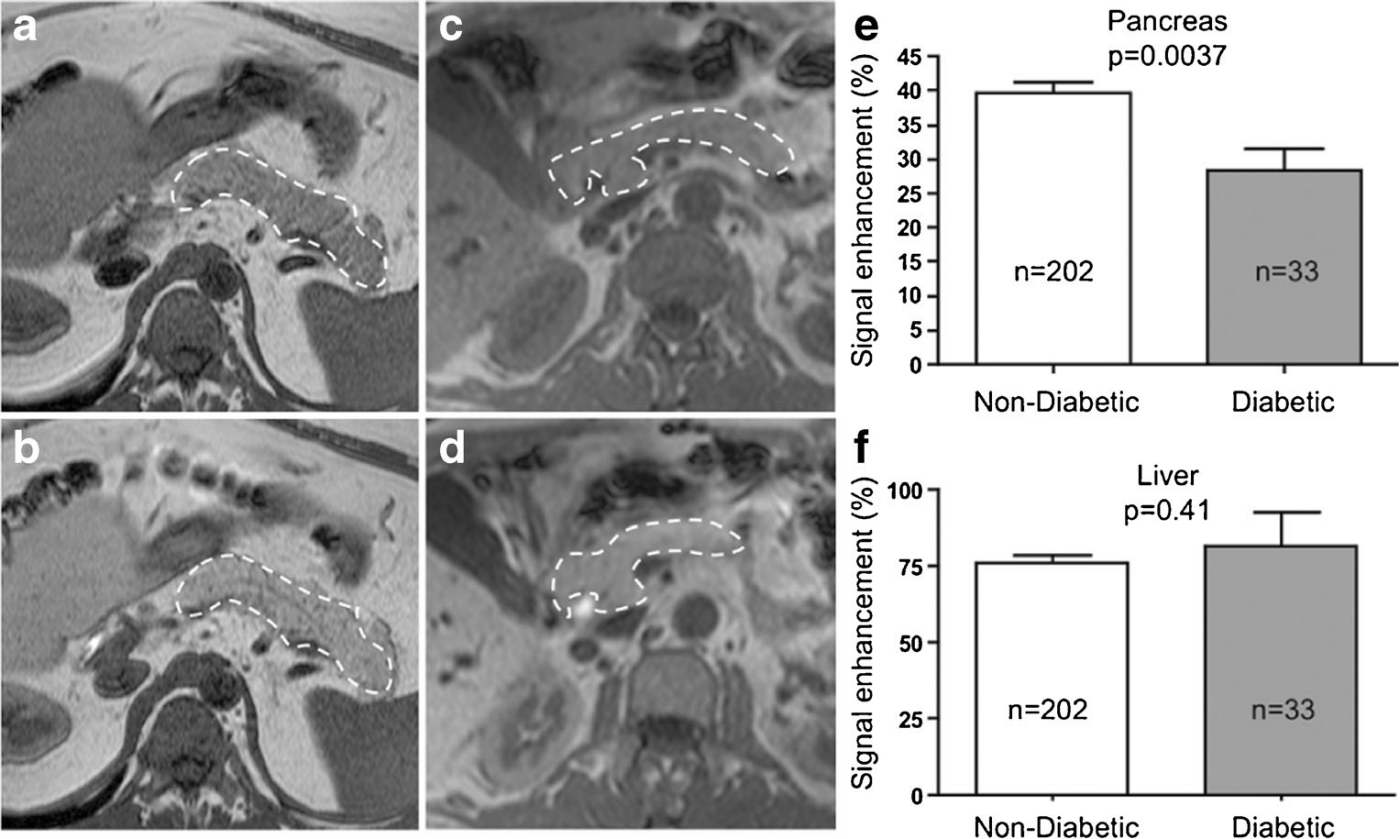
Mn^2+^-enhanced MRI of the pancreas (*dashed lines*). **a**, **c** Diabetic patient. **b**, **d** Normoglycemic patient. **e** Signal enchantment was significantly higher in normoglycemic patients [47]

Many studies have been published on manganese as a contrast agent for β-cell imaging. Mn^2+^ is a Ca^2+^ analogue that can enter β-cells trough voltage-gated Ca^2+^ channels, resulting in a specific, glucose-dependant signal [45, 118]. Administering 47 mg/kg of MnCl_2_ as a contrast agent, Lamprianou et al. were able to visualize single islets up to the size of 50 μm both ex vivo and in vivo, proving the feasibility of Mn^2+^ as a contrast agent for imaging native β-cells [44]. Antkowiak et al. were able to observe a gradual decrease of Mn^2+^ accumulation after cyclophosphamide-induced β-cell apoptosis, even before diabetic symptoms occurred, indicating the potential as a diagnostic tool for the early diagnosis of diabetes [45]. Dhyani et al. hypothesized that autoimmune damage to β-cells as well as changes in the microvasculature can predict the development of type 1 diabetes. Using Mn^2+^-enhanced dynamic MRI and an empirical mathematical model, they were able to observe different uptake in head, body, and tail of the pancreas in both healthy and diabetic mice. Following this data, they were able to link changes in β-cell and perfusion to functional transformations in the pathogenesis of diabetes, concluding that those parameters might be used as potential biomarkers for the diagnosis of diabetes [46]. A retrospective study by Bostikas et al. compiled the existing data from previous Mn^2+^-enhanced MRI data and assessed uptake in pancreas of both non-diabetic and type 2 diabetic patients, as indicated in Fig. 7. In both cases, the MRI signal was enhanced, however, the extent of the enhancement significantly correlated with the BMI of the patients but not other factors like age or insulin requirement. As this was a retrospective study, the number of patients was limited, and the images taken were not optimized for imaging the pancreas. It would be interesting to see if further studies in healthy and diabetic patients can confirm this data [47]. In summary, these results lead to the conclusion that Mn^2+^ might be suitable for the longitudinal imaging of β-cell mass and function, however, further studies in humans are necessary. In addition, toxicity of Mn^2+^ has to be investigated more closely before it can be routinely used in humans.

### Optical imaging

**Fig. 8:**
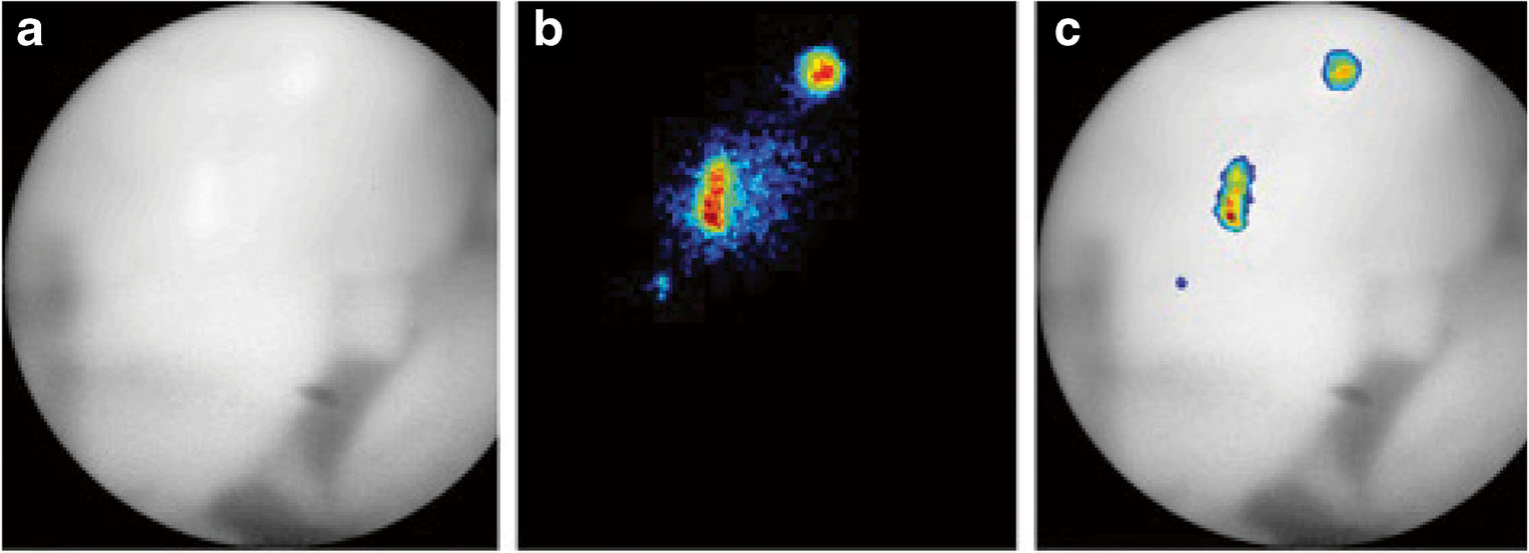
In vivo fluorescent microscopy of pancreatic islets in a mouse. **a** White light image. **b** Fluorescent image. **c** Combined picture. Adapted from [70]

Optical imaging combines both a very high spatial resolution (which can be high enough to image single cells) and a high sensitivity, making it a very valuable imaging modality. The first selective fluorescent probe targeting β-cells in vivo was developed in 2010 by Reiner et al. This first-generation fluorescent tracer was based on exendin-4 and targeted the GLP-1R. The fluorophore VT680 attached to K12 of exendin-4 bound to the receptor with high affinity and successfully imaged in a proof-of-concept study single islets in mice with intravital confocal microscopy demonstrating a quick and specific accumulation in β-cells. The fluorophore used in that study, however, did not have ideal properties, as the emission at 680 nm has a low tissue penetration. A second-generation probe developed in the same group utilized a fluorochrome emitting at 750 nm that circumvented tissue autofluorescence and increased tissue penetration. The accumulation also correlated with BCM in healthy and streptozotocin-induced diabetic mice and detected β-cells loss before diabetic symptoms appeared (Fig. 8) [68, 70]. Intravital confocal microscopy, however, is still an invasive method, and further studies are necessary to determine if this probe can be used non-invasively. A more recent study tested a bimodal PET/fluorescence tracer based on exendin-4 labeled with ^64^Cu, allowing both whole-body PET images and high-resolution images of single islets. The PET images successfully visualized the tumor in mouse xenografts, though, no in vivo fluorescent images have been performed so far [73].

**Fig. 9:**
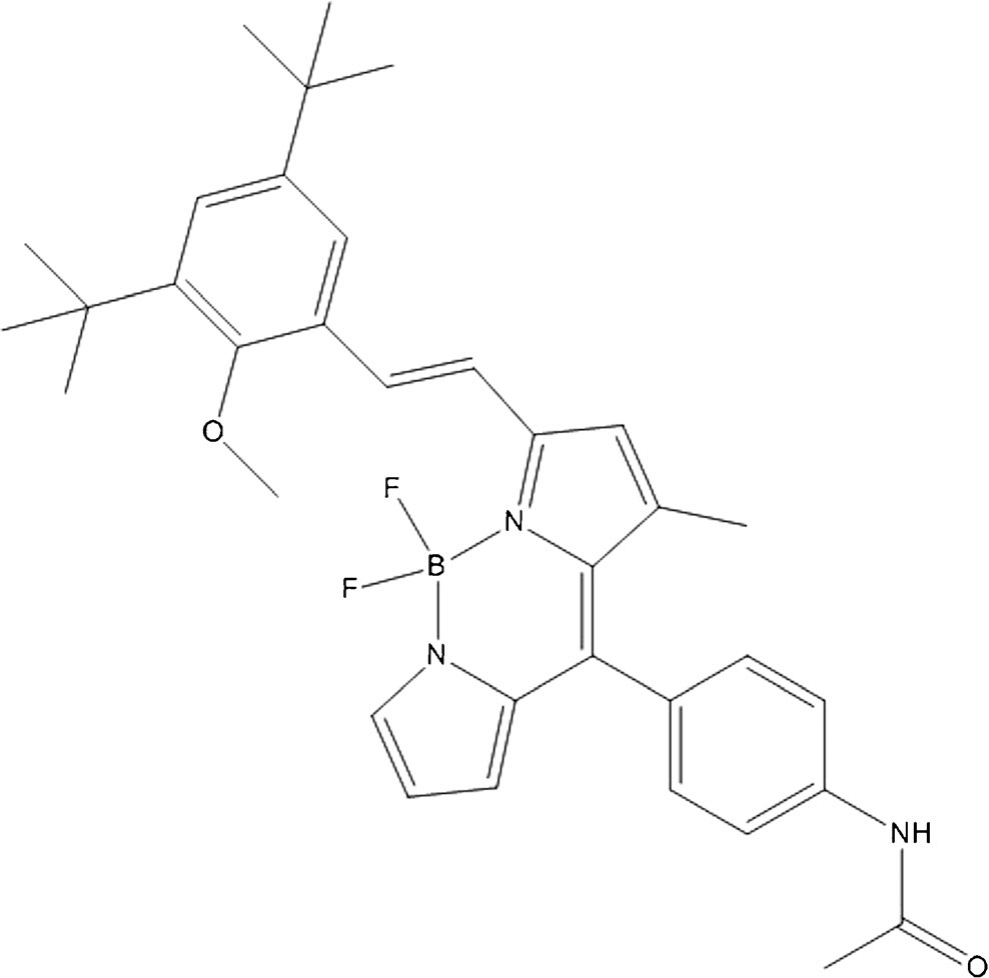
Molecular structure of PiY

Kang et al. chose a different approach by developing the novel small molecule PiY (Fig. 9), which after intravenous administration specifically labeled β-cells in the pancreas in both mice and rats. PiY also successfully distinguished between healthy and diabetic tissue, proving its feasibility for β-cell imaging. The specificity does not extend to organs such as lung or liver, although heart showed a higher uptake than the pancreas. Cellular uptake of the probe does not affect islet function and viability, which is important for longitudinal studies. Some aspects of PiY are still not fully clear, such as the molecular target of the probe, which has to be investigated to understand the selective staining of the β-cells. Another open question is if the probe is suitable for in vivo imaging use as all imaging studies so far were performed on ex vivo organ sections [48].

## Conclusions and future perspectives

Non-invasive imaging of β-cells remains a challenge. Even in the healthy pancreas, an organ with 100–150 g, only a low number of β-cells are present, merely 1–2% of the pancreas are β-cells, which in addition are located close to intestinal organs that can accumulate potential tracers and thus obstructing the specific signal [119]. So far, many various approaches for different applications have been tested. The most promising application is the visualization of insulinomas and β-cell hyperplasia. As they usually consist of β-cell clusters, accumulation in those foci is usually stronger, allowing specific localization of those lesions. Several radiotracers, both for PET and SPECT imaging, are currently being used in clinics. ^18^F-DOPA, [^11^C]-5-HTP, and various exendin-based probes have been successfully tested so far [33, 34, 51, 98]. Additionally, most of the β-cell-specific probes allow the assessment of transplanted islets [12, 14, 31, 113–116].

The accurate quantification of BCM via imaging, however, remains elusive. Although several groups reported changes in tracer uptake of a diabetic pancreas as compared to healthy organs in animal models and humans, several important factors have to be considered [22, 67, 70]. First of all, the tracer has to be specific for β-cells. Residual uptake in other islet cells or the exocrine pancreas can result in an overestimation of the BCM [23, 89]. Secondly, the variability of BCM in healthy patients is very high, meaning that longitudinal measurements are necessary to observe changes in BCM over a period of time [66, 67]. This, however, means that the tracer has to be non-toxic and should not damage either β-cells or other tissue. This potentially might be a problem for Mn^2+^ as MRI contrast agent, because the toxicity has not yet fully tested, and radiotracers that accumulate in non-target tissues leading to a high radiation burden for the patients. Lastly, PET and SPECT display a partial volume effect if the signal derives from an object that is smaller than the spatial resolution of the scanner, which may lead to an underestimation of the signal. Unfortunately, until now, none of the currently available tracers or contrast agents have been able to address all challenges.

The most promising target so far is the GLP-1R. Various studies with derivatives of exendin, the lead structure for GLP-1R ligands, proved that the peptide can be modified with various moieties without altering the biological properties. This emphasizes the versatility of this peptide, which has been successfully used as a probe for PET, SPECT, MRI, and optical imaging [43, 117, 120]. A recent study showing that exendin-4 can be modified in various positions without losing the affinity to the receptor concludes that multiple moieties might be attached to the peptide, allowing a wider variety of tracers [69]. Such modifications might include multiple chelators, allowing an increase of the specific activity of the tracer or the conjugation of two different functionalities, for instance, a radioactive moiety and an optical dye. This might allow a preoperative assessment of the lesions using PET as well as direct visualization of the small foci during the surgery. A dual-imaging probe for this approach, based on exendin-4, has recently been published, and it is an important step towards new hybrid imaging techniques for use in the clinic [73].

Probes containing optical dyes have a high potential for future application. Guided surgery can allow a more precise identification of the lesions during the operation and the high sensitivity of the fluorescent probes allows the visualization of the smallest lesions.

In conclusion, many different probes have been tested for imaging of β-cells, however, each tracer and imaging modalities have different drawbacks. The most promising target so far is the GLP-1R, as it is specifically expressed on β-cells and exendin-4, a specific agonist of the receptor that can be modified at various positions without losing the biological properties. Thus, it can be utilized to attach various functional groups for different imaging modalities allowing a high degree of flexibility for clinical use.

## References

[1] Atkinson MA. The pathogenesis and natural history of type 1 diabetes. Cold Spring Harb Perspect Med. 2012;2(11). 10.1101/cshperspect.a007641 PMC3543105 23125199

[2] Elayat AA, el-Naggar MM, Tahir M (1995). An immunocytochemical and morphometric study of the rat pancreatic islets. J Anat.

[3] Wang X, Misawa R, Zielinski MC, Cowen P, Jo J, Periwal V, et al. Regional differences in islet distribution in the human pancreas—preferential beta-cell loss in the head region in patients with type 2 diabetes. Kay TWH, editor. PLoS ONE. 2013;8(6):e67454. 10.1371/journal.pone.0067454 PMC3691162 23826303

[4] Ratner RE (2001). Glycemic control in the prevention of diabetic complications. Clin Cornerstone.

[5] Lacherade J-C, Jacqueminet S, Preiser J-C (2009). An overview of hypoglycemia in the critically ill. J Diabetes Sci Technol.

[6] Danaei G, Finucane MM, Lu Y, Singh GM, Cowan MJ, Paciorek CJ (2011). National, regional, and global trends in fasting plasma glucose and diabetes prevalence since 1980: systematic analysis of health examination surveys and epidemiological studies with 370 country-years and 2 · 7 million participants. Lancet.

[7] van Belle TL, Coppieters KT, von Herrath MG (2011). Type 1 diabetes: etiology, immunology, and therapeutic strategies. Physiol Rev.

[8] Caro JF, Dohm LG, Pories WJ, Sinha MK (1989). Cellular alterations in liver, skeletal muscle, and adipose tissue responsible for insulin resistance in obesity and type II diabetes. Diabetes Metab Rev.

[9] Wilmot E, Idris I (2014). Early onset type 2 diabetes: risk factors, clinical impact and management. Ther Adv Chronic Dis.

[10] Sreenan S, Pick AJ, Levisetti M, Baldwin AC, Pugh W, Polonsky KS (1999). Increased β-cell proliferation and reduced mass before diabetes onset in the nonobese diabetic mouse. Diabetes Am Diabetes Assoc.

[11] DeFronzo RA (2009). From the triumvirate to the ominous octet: a new paradigm for the treatment of type 2 diabetes mellitus. Diabetes.

[12] Pattou F, Kerr-Conte J, Wild D (2010). GLP-1-receptor scanning for imaging of human beta cells transplanted in muscle. N Engl J Med.

[13] Dixon S, Tapping CR, Walker JN, Bratby M, Anthony S, Boardman P (2012). The role of interventional radiology and imaging in pancreatic islet cell transplantation. Clin Radiol.

[14] Kriz J, Jirak D, Berkova Z, Herynek V, Lodererova A, Girman P (2011). Detection of pancreatic islet allograft impairment in advance of functional failure using magnetic resonance imaging. Transplant Int.

[15] Arya V, Mohammed Z, Blankenstein O, De Lonlay P, Hussain K (2014). Hyperinsulinaemic hypoglycaemia. Horm Metab Res.

[16] Raffel A, Krausch MM, Anlauf M, Wieben D, Braunstein S, Klöppel G (2007). Diffuse nesidioblastosis as a cause of hyperinsulinemic hypoglycemia in adults: a diagnostic and therapeutic challenge. Surgery.

[17] Shin JJ, Gorden P, Libutti SK (2010). Insulinoma: pathophysiology, localization and management. Future Oncol.

[18] Boukhman MP, Karam JM, Shaver J, Siperstein AE, DeLorimier AA, Clark OH (1999). Localization of insulinomas. Arch Surg.

[19] Fagerholm V, Mikkola KK, Ishizu T, Arponen E, Kauhanen S, Nagren K (2010). Assessment of islet specificity of dihydrotetrabenazine radiotracer binding in rat pancreas and human pancreas. J Nucl Med.

[20] Jahan M, Eriksson O, Johnström P, Korsgren O, Sundin A, Johansson L, et al. Decreased defluorination using the novel beta-cell imaging agent [^18^F]FE-DTBZ-d4 in pigs examined by PET. EJNMMI Research. Springer Open Ltd; 2011;1(1):33. 10.1186/2191-219X-1-33 PMC3284452 22214308

[21] Eriksson O, Jahan M, Johnström P, Korsgren O, Sundin A, Halldin C (2010). In vivo and in vitro characterization of [^18^F]-FE-(+)-DTBZ as a tracer for beta-cell mass. Nucl Med Biol.

[22] Normandin MD, Petersen KF, Ding YS, Lin SF, Naik S, Fowles K (2012). In vivo imaging of endogenous pancreatic β-cell mass in healthy and type 1 diabetic subjects using ^18^F-Fluoropropyl-Dihydrotetrabenazine and PET. J Nucl Med.

[23] Harris PE, Farwell MD, Ichise M (2013). PET quantification of pancreatic VMAT 2 binding using (+) and (−) enantiomers of [^18^F]FP-DTBZ in baboons. Nucl Med Biol.

[24] Schneider S, Feilen PJ, Schreckenberger M, Schwanstecher M, Schwanstecher C, Buchholz HG (2005). In vitro and in vivo evaluation of novel glibenclamide derivatives as imaging agents for the non-invasive assessment of the pancreatic islet cell mass in animals and humans. Exp Clin Endocrinol Diabetes.

[25] Oh C-S, Kohanim S, Kong F-L, Song H-C, Huynh N, Mendez R (2012). Sulfonylurea receptor as a target for molecular imaging of pancreas beta cells with ^99m^Tc-DTPA-glipizide. Ann Nucl Med.

[26] Kavishwar A, Medarova Z, Moore A (2011). Unique sphingomyelin patches are targets of a beta-cell-specific antibody. J Lipid Res.

[27] Moore A (2009). Advances in beta-cell imaging. Eur J Radiol.

[28] Ueberberg S, Meier JJ, Waengler C, Schechinger W, Dietrich JW, Tannapfel A (2009). Generation of novel single-chain antibodies by phage-display technology to direct imaging agents highly selective to pancreatic β- or α-cells in vivo. Diabetes.

[29] Balla DZ, Gottschalk S, Shajan G, Ueberberg S, Schneider S, Hardtke-Wolenski M, et al. In vivo visualization of single native pancreatic islets in the mouse. Aime S, Muller RN, editors. Contrast Media Mol. Imaging. 2013;8(6):495–504. 10.1002/cmmi.1580 24375905

[30] Vats D, Wang H, Esterhazy D, Dikaiou K, Danzer C, Honer M (2012). Multimodal imaging of pancreatic beta cells in vivo by targeting transmembrane protein 27 (TMEM27). Diabetologia.

[31] Eriksson O, Eich T, Sundin A, Tibell A, Tufveson G, Andersson H (2009). Positron emission tomography in clinical islet transplantation. Am J Transplant.

[32] Malaisse WJ, Damhaut P, Malaisse-Lagae F, Ladriere L, Olivares E, Goldman S (2000). Fate of 2-deoxy-2-[^18^F]fluoro-d-glucose in control and diabetic rats. Int J Mol Med.

[33] Imperiale A, Sebag F, Vix M, Castinetti F, Kessler L, Moreau F (2015). ^18^F-FDOPA PET/CT imaging of insulinoma revisited. Eur J Nucl Med Mol Imaging.

[34] Gopal-Kothandapani JS, Hussain K (2014). Congenital hyperinsulinism: role of fluorine-18L-3, 4 hydroxyphenylalanine positron emission tomography scanning. World J Radiol.

[35] Kauhanen S, Seppänen M, Minn H, Nuutila P (2010). Clinical PET imaging of insulinoma and beta-cell hyperplasia. Curr Pharm Des.

[36] Eriksson O, Espes D, Selvaraju RK, Jansson E, Antoni G, Sorensen J (2014). Positron emission tomography Ligand [^11^C]5-Hydroxy-tryptophan can be used as a surrogate marker for the human endocrine pancreas. Diabetes.

[37] Di Gialleonardo V, de Vries EFJ, Di Girolamo M, Quintero AM, Dierckx RAJO, Signore A (2012). Imaging of β-cell mass and insulitis in insulin-dependent (type 1) diabetes mellitus. Endocr Rev.

[38] Rubí B, Ljubicic S, Pournourmohammadi S, Carobbio S, Armanet M, Bartley C (2005). Dopamine D2-like receptors are expressed in pancreatic beta cells and mediate inhibition of insulin secretion. J Biol Chem.

[39] Garcia A, Mirbolooki MR, Constantinescu C, Pan ML, Sevrioukov E, Milne N (2011). ^18^F-Fallypride PET of pancreatic islets: in vitro and in vivo rodent studies. J Nucl Med.

[40] Garcia A, Venugopal A, Pan M-L, Mukherjee J (2014). Imaging pancreas in healthy and diabetic rodent model using [^18^F]fallypride positron emission tomography/computed tomography. Diabetes Technol Ther.

[41] Lv J, Pan Y, Li X, Cheng D, Liu S, Shi H, et al. The imaging of insulinomas using a radionuclide-labelled molecule of the GLP-1 analogue liraglutide: a new application of liraglutide. Holscher C, editor. PLoS ONE. 2014;9(5):e96833. 10.1371/journal.pone.0096833 PMC4013070 24805918

[42] Lubag AJ, De Leon-Rodriguez LM, Burgess SC, Sherry AD (2011). Noninvasive MRI of β-cell function using a Zn^2+^-responsive contrast agent. Proc Natl Acad Sci U S A.

[43] Wild D, Wicki A, Mansi R, Behe M, Keil B, Bernhardt P (2010). Exendin-4-based radiopharmaceuticals for glucagon-like peptide-1 receptor PET/CT and SPECT/CT. J Nucl Med.

[44] Lamprianou S, Immonen R, Nabuurs C, Gjinovci A, Vinet L, Montet XCR (2011). High-resolution magnetic resonance imaging quantitatively detects individual pancreatic islets. Diabetes.

[45] Antkowiak PF, Vandsburger MH, Epstein FH (2011). Quantitative pancreatic β cell MRI using manganese-enhanced look-locker imaging and two-site water exchange analysis. Magn Reson Med.

[46] Dhyani AH, Fan X, Leoni L, Haque M, Roman BB. Empirical mathematical model for dynamic manganese-enhanced MRI of the murine pancreas for assessment of β-cell function. Magn Reson Imaging. Elsevier Inc; 2013;31(4):508–14. 10.1016/j.mri.2012.09.003 PMC3582819 23102946

[47] Botsikas D, Terraz S, Vinet L, Lamprianou S, Becker CD, Bosco D (2012). Pancreatic magnetic resonance imaging after manganese injection distinguishes type 2 diabetic and normoglycemic patients. Islets.

[48] Kang N-Y, Lee S-C, Park S-J, Ha H-H, Yun S-W, Kostromina E (2013). Visualization and isolation of Langerhans islets by a fluorescent probe PiY. Angew Chem Int Ed.

[49] Wicki A, Wild D, Storch D, Seemayer C, Gotthardt M, Béhé M, et al. [Lys^40^ (Ahx-DTPA-^111^In) NH_2_]-Exendin-4 is a highly efficient radiotherapeutic for glucagon-like peptide-1 receptor–targeted therapy for insulinoma. Clin Cancer Res. 2007;13(12):3696–705. 10.1158/1078-0432.CCR-06-2965 17575235

[50] Wild D, Béhé M, Wicki A, Storch D, Waser B, Gotthardt M (2006). [Lys^40^(Ahx-DTPA-^111^In)NH_2_]-exendin-4, a very promising ligand for glucagon-like peptide-1 (GLP-1) receptor targeting. J Nucl Med.

[51] Christ E, Wild D, Ederer S, Behe M, Nicolas G (2013). Glucagon-like peptide-1 receptor imaging for the localisation of insulinomas: a prospective multicentre imaging study. Lancet Diabetes Endocrinol.

[52] Christ E, Wild D, Forrer F, Brändle M, Sahli R, Clerici T (2009). Glucagon-like peptide-1 receptor imaging for localization of insulinomas. J Clin Endocrinol Metab.

[53] Sowa-Staszczak A, Pach D, Mikołajczak R, Mäcke H, Jabrocka-Hybel A, Stefańska A (2013). Glucagon-like peptide-1 receptor imaging with [Lys^40^(Ahx-HYNIC- ^99m^Tc/EDDA)NH_2_]-exendin-4 for the detection of insulinoma. Eur J Nucl Med Mol Imaging.

[54] Selvaraju RK, Velikyan I, Johansson L, Wu Z, Todorov I, Shively J (2013). In vivo imaging of the glucagon-like peptide 1 receptor in the pancreas with ^68^Ga-labeled DO3A-exendin-4. J Nucl Med.

[55] Wu Z, Todorov I, Li L, Bading JR, Li Z, Nair I (2011). In vivo imaging of transplanted islets with ^64^Cu-DO3A-VS-Cys^40^-exendin-4 by targeting GLP-1 receptor. Bioconjugate Chem.

[56] Mikkola K, Kirsi M, Yim C-B, Cheng-Bin Y, Fagerholm V, Veronica F (2014). 64Cu- and 68Ga-labelled [Nle14, Lys40(Ahx-NODAGA)NH2]-exendin-4 for pancreatic beta cell imaging in rats. Mol Imaging Biol.

[57] Kiesewetter DO, Gao H, Ma Y, Niu G, Quan Q, Guo N (2012). ^18^F-radiolabeled analogs of exendin-4 for PET imaging of GLP-1 in insulinoma. Eur J Nucl Med Mol Imaging.

[58] Wu Z, Liu S, Hassink M, Nair I, Park R, Li L (2013). Development and evaluation of ^18^F-TTCO-Cys^40^-exendin-4: a PET probe for imaging transplanted islets. J Nucl Med.

[59] Keliher EJ, Reiner T, Thurber GM, Upadhyay R, Weissleder R (2012). Efficient ^18^F-labeling of synthetic exendin-4 analogues for imaging beta cells. Chem Open.

[60] Yim C-B, Mikkola K, Fagerholm V, Elomaa V-V, Ishizu T, Rajander J (2013). Synthesis and preclinical characterization of [^64^Cu]NODAGA-MAL-exendin-4 with a* N*^ε^-maleoyl-l-lysyl-glycine linkage. Nucl Med Biol.

[61] Kiesewetter DO, Guo N, Guo J, Gao H, Zhu L, Ma Y (2012). Evaluation of an [^18^F]AlF-NOTA analog of exendin-4 for imaging of GLP-1 receptor in insulinoma. Theranostics.

[62] Waser B, Reubi JC (2011). Value of the radiolabelled GLP-1 receptor antagonist exendin(9–39) for targeting of GLP-1 receptor-expressing pancreatic tissues in mice and humans. Eur J Nucl Med Mol Imaging.

[63] Waser B, Reubi JC (2014). Radiolabelled GLP-1 receptor antagonist binds to GLP-1 receptor-expressing human tissues. Eur J Nucl Med Mol Imaging.

[64] Gotthardt M, Lalyko G, van Eerd-Vismale J, Keil B, Schurrat T, Hower M (2006). A new technique for in vivo imaging of specific GLP-1 binding sites: first results in small rodents. Regul Pept.

[65] Brom M, Oyen WJG, Joosten L, Gotthardt M, Boerman OC (2010). ^68^Ga-labelled exendin-3, a new agent for the detection of insulinomas with PET. Eur J Nucl Med Mol Imaging.

[66] Brom M, Joosten L, Oyen WJG, Gotthardt M, Boerman OC (2012). Radiolabelled GLP-1 analogues for in vivo targeting of insulinomas. Contrast Media Mol Imaging.

[67] Brom M, Woliner-van der Weg W, Joosten L, Frielink C, Bouckenooghe T, Rijken P (2014). Non-invasive quantification of the beta cell mass by SPECT with ^111^In-labelled exendin. Diabetologia.

[68] Reiner T, Kohler RH, Liew CW, Hill JA, Gaglia J, Kulkarni RN (2010). Near-infrared fluorescent probe for imaging of pancreatic β cells. Bioconjugate Chem.

[69] Jodal A, Lankat-Buttgereit B, Brom M, Schibli R, Béhé M (2014). A comparison of three ^67/68^Ga-labelled exendin-4 derivatives for β-cell imaging on the GLP-1 receptor: the influence of the conjugation site of NODAGA as chelator. EJNMMI Res.

[70] Reiner T, Thurber G, Gaglia J, Vinegoni C, Liew CW, Upadhyay R (2011). Accurate measurement of pancreatic islet β-cell mass using a second-generation fluorescent exendin-4 analog. Proc Natl Acad Sci U S A.

[71] Jodal A, Pape F, Becker-Pauly C, Maas O, Schibli R, Béhé M. Evaluation of ^111^In-labelled exendin-4 derivatives containing different Meprin β-specific cleavable linkers. Cebecauer M, editor. PLoS ONE. 2015;10(4):e0123443. 10.1371/journal.pone.0123443 PMC4391719 25855967

[72] Vinet L, Lamprianou S, Babič A, Lange N, Thorel F, Herrera PL (2015). Targeting GLP-1 receptors for repeated magnetic resonance imaging differentiates graded losses of pancreatic beta cells in mice. Diabetologia.

[73] Brand C, Abdel-Atti D, Zhang Y, Carlin S, Clardy SM, Keliher EJ (2014). In vivo imaging of GLP-1R with a targeted bimodal PET/fluorescence imaging agent. Bioconjugate Chem.

[74] Henderson RG (1983). Nuclear magnetic resonance imaging: a review. J R Soc Med.

[75] Massoud TF (2003). Molecular imaging in living subjects: seeing fundamental biological processes in a new light. Genes Dev.

[76] Culver J, Akers W, Achilefu S (2008). Multimodality molecular imaging with combined optical and SPECT/PET modalities. J Nucl Med.

[77] Shokrollahi H. Contrast agents for MRI. Mater Sci Eng C Mater Biol Appl. Elsevier B.V; 2013;33(8):4485–97. 10.1016/j.msec.2013.07.012 24094150

[78] Bruyant PP (2002). Analytic and iterative reconstruction algorithms in SPECT. J Nucl Med.

[79] Mariani G, Bruselli L, Kuwert T, Kim EE, Flotats A, Israel O (2010). A review on the clinical uses of SPECT/CT. Eur J Nucl Med Mol Imaging.

[80] Ter-Pogossian MM, Phelps ME, Hoffman EJ, Mullani NA (1975). A positron-emission transaxial tomograph for nuclear imaging (PETT). Radiology.

[81] Czernin J, Benz MR, Allen-Auerbach MS (2009). PET imaging of prostate cancer using ^11^C-acetate. PET Clin.

[82] Shetty D, Lee Y-S, Jeong JM (2010). ^68^Ga-labeled radiopharmaceuticals for positron emission tomography. Nucl Med Mol Imaging.

[83] Parry JJ, Andrews R, Rogers BE (2006). MicroPET imaging of breast cancer using radiolabeled bombesin analogs targeting the gastrin-releasing peptide receptor. Breast Cancer Res Treat.

[84] Razansky D, Deliolanis NC, Vinegoni C, Ntziachristos V (2012). Deep tissue optical and optoacoustic molecular imaging technologies for pre-clinical research and drug discovery. Curr Pharm Biotechnol.

[85] Sekiguchi Y, Owada J, Oishi H, Katsumata T, Ikeda K, Kudo T (2012). Noninvasive monitoring of β-cell mass and fetal β-cell genesis in mice using bioluminescence imaging. Exp Anim.

[86] Choy G, Choyke P, Libutti SK (2003). Current advances in molecular imaging: noninvasive in vivo bioluminescent and fluorescent optical imaging in cancer research. Mol Imaging.

[87] Arifin DR, Bulte JWM. Imaging of pancreatic islet cells. Park Y, Kim K, Pozzilli P, editors. Diabetes Metab Res Rev. 2011;27(8):761–6. 10.1002/dmrr.1248 PMC3218557 22069256

[88] Liu Z, Miller SJ, Joshi BP, Wang TD (2013). In vivo targeting of colonic dysplasia on fluorescence endoscopy with near-infrared octapeptide. Gut.

[89] Saisho Y, Harris PE, Butler AE, Galasso R, Gurlo T, Rizza RA (2008). Relationship between pancreatic vesicular monoamine transporter 2 (VMAT2) and insulin expression in human pancreas. J Mol Histol.

[90] Watanabe M, Takemura H, Mizoguchi H, Hyodo H, Soga K, Zako T (2014). Development of novel endoscope with NIR camera using real-time video composite method. IFMBE proceedings.

[91] Anlauf M, Eissele R, Schäfer MKH, Eiden LE, Arnold R, Pauser U (2003). Expression of the two isoforms of the vesicular monoamine transporter (VMAT1 and VMAT2) in the endocrine pancreas and pancreatic endocrine tumors. J Histochem Cytochem.

[92] Eiden LE, Schäfer MK-H, Weihe E, Schütz B (2004). The vesicular amine transporter family (SLC18): amine/proton antiporters required for vesicular accumulation and regulated exocytotic secretion of monoamines and acetylcholine. Pflugers Arch.

[93] Kung MP, Hou C, Lieberman BP, Oya S, Ponde DE, Blankemeyer E (2008). In vivo imaging of β-cell mass in rats using ^18^F-FP-(+)-DTBZ: a potential PET ligand for studying diabetes mellitus. J Nucl Med.

[94] Singhal T, Ding Y-S, Weinzimmer D, Normandin MD, Labaree D, Ropchan J (2010). Pancreatic beta cell mass PET imaging and quantification with [^11^C]DTBZ and [^18^F]FP-(+)-DTBZ in rodent models of diabetes. Mol Imaging Biol.

[95] Schäfer MKH, Hartwig NR, Kalmbach N, Klietz M, Anlauf M, Eiden LE (2013). Species-specific vesicular monoamine transporter 2 (VMAT2) expression in mammalian pancreatic beta cells: implications for optimising radioligand-based human beta cell mass (BCM) imaging in animal models. Diabetologia.

[96] Proks P, Reimann F, Green N, Gribble F, Ashcroft F (2002). Sulfonylurea stimulation of insulin secretion. Diabetes.

[97] Moore A, Bonner-Weir S, Weissleder R (2001). Noninvasive in vivo measurement of beta-cell mass in mouse model of diabetes. Diabetes.

[98] Tessonnier L, Sebag F, Ghander C, De Micco C, Reynaud R, Palazzo FF (2010). Limited value of ^18^F-F-DOPA PET to localize pancreatic insulin-secreting tumors in adults with hyperinsulinemic hypoglycemia. J Clin Endocrinol Metab.

[99] Akizawa H, Arano Y, Mifune M, Iwado A, Saito Y, Mukai T (2001). Effect of molecular charges on renal uptake of ^111^In-DTPA-conjugated peptides. Nucl Med Biol.

[100] Bird JL, Wright EE, Feldman JM (1980). Pancreatic islets: a tissue rich in serotonin. Diabetes.

[101] Ohta Y, Kosaka Y, Kishimoto N, Wang J, Smith SB, Honig G (2011). Convergence of the insulin and serotonin programs in the pancreatic β-cell. Diabetes.

[102] Tornehave D, Kristensen P, Romer J, Knudsen LB, Heller RS (2008). Expression of the GLP-1 receptor in mouse, rat, and human pancreas. J Histochem Cytochem.

[103] Kolligs F, Fehmann HC, Göke R, Göke B (1995). Reduction of the incretin effect in rats by the glucagon-like peptide 1 receptor antagonist exendin (9–39) amide. Diabetes.

[104] Wang Z, Wang RM, Owji AA, Smith DM, Ghatei MA, Bloom SR (1995). Glucagon-like peptide-1 is a physiological incretin in rat. J Clin Invest.

[105] Fehmann HC, Habener JF (1992). Insulinotropic hormone glucagon-like peptide-I(7–37) stimulation of proinsulin gene expression and proinsulin biosynthesis in insulinoma beta TC-1 cells. Endocrinology.

[106] Stoffers DA, Kieffer TJ, Hussain MA, Drucker DJ, Bonner-Weir S, Habener JF (2000). Insulinotropic glucagon-like peptide 1 agonists stimulate expression of homeodomain protein IDX-1 and increase islet size in mouse pancreas. Diabetes.

[107] Xu G, Stoffers DA, Habener JF, Bonner-Weir S (1999). Exendin-4 stimulates both beta-cell replication and neogenesis, resulting in increased beta-cell mass and improved glucose tolerance in diabetic rats. Diabetes.

[108] Deacon CF, Johnsen AH, Holst JJ (1995). Degradation of glucagon-like peptide-1 by human plasma in vitro yields an N-terminally truncated peptide that is a major endogenous metabolite in vivo. J Clin Endocrinol Metabol.

[109] Wang Y, Lim K, Normandin M, Zhao X, Cline GW, Ding Y-S. Synthesis and evaluation of [^18^F]exendin (9–39) as a potential biomarker to measure pancreatic β-cell mass. Nucl Med Biol. Elsevier Inc. 2011; 1–10. 10.1016/j.nucmedbio.2011.07.011 PMC4484741 22033026

[110] Velikyan I, Bulenga TN, Selvaraju R, Lubberink M, Espes D, Rosenstrom U (2015). Dosimetry of [(177)Lu]-DO3A-VS-Cys(40)-exendin-4—impact on the feasibility of insulinoma internal radiotherapy. Am J Nucl Med Mol Imaging.

[111] Gotthardt M, van Eerd-Vismale J, Oyen WJG, De Jong M, Zhang H, Rolleman E (2007). Indication for different mechanisms of kidney uptake of radiolabeled peptides. J Nucl Med.

[112] Fujioka Y, Arano Y, Ono M, Uehara T, Ogawa K, Namba S (2001). Renal metabolism of 3′-iodohippuryl* N*ε-maleoyl-l-lysine (HML)-conjugated fab fragments. Bioconjugate Chem.

[113] Zhang B, Jiang B, Chen Y, Huang H, Xie Q, Kang M (2012). Detection of viability of transplanted beta cells labeled with a novel contrast agent - polyvinylpyrrolidone-coated superparamagnetic iron oxide nanoparticles by magnetic resonance imaging. Contrast Media Mol Imaging.

[114] Oishi K, Miyamoto Y, Saito H, Murase K, Ono K, Sawada M, et al. In vivo imaging of transplanted islets labeled with a novel cationic nanoparticle. Rozhkova EA, editor. PLoS ONE. 2013;8(2):e57046. 10.1371/journal.pone.0057046 PMC3579774 23451139

[115] Lee N, Kim H, Choi SH, Park M, Kim D, Kim HC (2011). Magnetosome-like ferrimagnetic iron oxide nanocubes for highly sensitive MRI of single cells and transplanted pancreatic islets. Proc Natl Acad Sci U S A.

[116] Zacharovová K, Berková Z, Jirák D, Herynek V, Vancová M, Dovolilová E (2012). Processing of superparamagnetic iron contrast agent ferucarbotran in transplanted pancreatic islets. Contrast Media Mol Imaging.

[117] Wang P, Yoo B, Yang J, Zhang X, Ross A, Pantazopoulos P (2014). GLP-1R-targeting magnetic nanoparticles for pancreatic islet imaging. Diabetes.

[118] Leoni L, Dhyani A, La Riviere P, Vogt S, Lai B, Roman BB (2011). β-Cell subcellular localization of glucose-stimulated Mn uptake by X-ray fluorescence microscopy: implications for pancreatic MRI. Contrast Media Mol Imaging.

[119] In’t Veld P, Marichal M (2010). Microscopic anatomy of the human islet of Langerhans. Adv Exp Med Biol.

[120] Clardy SM, Keliher EJ, Mohan JF, Sebas M, Benoist C, Mathis D (2014). Fluorescent exendin-4 derivatives for pancreatic β-cell analysis. Bioconjugate Chem.

